# Patterns of Communication Technology Utilization for Health Information Among Hispanics in South Carolina: Implications for Health Equity

**DOI:** 10.1089/heq.2016.0013

**Published:** 2017-01-01

**Authors:** DeAnne K. Hilfinger Messias, Robin Dawson Estrada

**Affiliations:** ^1^ College of Nursing and Women's and Gender Studies Program, University of South Carolina, Columbia, South Carolina.; ^2^ College of Nursing, University of South Caroline, Columbia, South Carolina.

**Keywords:** Hispanic health, information-seeking, technology

## Abstract

**Background:** Language, culture, geographic, social, and economic factors are associated with health disparities. Among more recent Hispanic immigrants, limited English proficiency and immigration status are barriers to health information and healthcare access. Improved access to culturally and linguistically tailored health information through technology could potentially enhance healthcare access and health outcomes. However, little is known about health information-seeking through technology among Hispanics in recent settlement areas.

**Purpose:** The aim of this exploratory study was to describe patterns of self-reported utilization of technology for health information-seeking among the growing Hispanic population in South Carolina (SC) over a period of 5 years.

**Methods:** Descriptive, community-based, cross-sectional survey of 361 Hispanic adults residing in SC, conducted in 2011 and 2015/2016.

**Results:** Reflective of reported national trends, self-reported accessibility and utilization of cellphones increased (89–96.6%) among this sample. Although computer ownership decreased (58–53.9%), internet utilization for health information-seeking increased (45–57.8%); more than 80% of participants indicated that they considered the internet a “good source of health information.” The majority of participants in both time periods conducted health information searches in Spanish, although the reported access to English-language information increased over time.

**Conclusions:** These findings illustrate the increasing access and utilization of technology for health information among Hispanics in SC, underscoring the need for broader dissemination of culturally and linguistically appropriate health information through accessible technology, including Spanish language websites. Recommendations for future research include examining relationships between technology access, health service access and utilization, and health behaviors among Hispanics in diverse geographic and social contexts.

## Introduction

Hispanics are the fastest growing population group in the United States, and despite increasing levels of healthcare access among Hispanic adults, significant disparities persist.^1,2^ Compared with non-Hispanic Whites, Blacks, and Asians, Hispanics are less likely to report having a regular source of healthcare services.^3^ Further, Hispanic immigrants are more likely than US-born Hispanics to encounter economic, social, linguistic, and cultural barriers in accessing healthcare services, including lack of health insurance, cost of care, documentation status, and language discordance,^4–9^ and they are less likely than members of other racial/ethnic groups to seek health information outside a formal healthcare encounter.^10^

Reflecting the premise that access to information is critical to timely and effective healthcare access, increasing the number of internet users who can easily access appropriate health information is among the Healthy People 2020 goals for achieving health equity.^11^ Access to health information on the internet through a range of devices (e.g., computers, cellphones, smartphones) is associated with increased patient knowledge and preventive healthcare services utilization; conversely, barriers to internet access are associated with poorer health outcomes.^12^ Factors contributing to this digital divide range from the economic costs of both devices and data plans to rurality, literacy and educational levels, and language discordance.^13,14^ Despite a narrowing digital divide in recent years, in terms of internet usage, immigrant and Spanish-language dominant Hispanics continue to lag behind Whites, US-born, and English-language dominant Hispanics.^15^ This digital divide also intersects with access to healthcare services and information. As of 2014, compared with non-Hispanic Whites and African Americans, Hispanics had the lowest group rate on measures of internet access related to health information.^11^ Hispanic women were less likely to access the internet than Whites and African Americans, associated primarily with lack of computer expertise^16^; and less than half of Hispanic safety net clinic patients seeking cancer information reported having internet access.^17^ However, with current cellphone ownership rate among urban-dwelling Hispanics at more than 90%,^18^ there are increasing opportunities for healthcare providers and systems to incorporate technology-based approaches that are aimed at reducing information access barriers, improving access to care, and ultimately enhancing health outcomes and decreasing health disparities.

Technology-based approaches that anticipate and address constraints and barriers related to language, culture, economics, and geography have the potential to mitigate health and healthcare disparities. There are national-level data on access to and utilization of technology (i.e., computers, internet, cellular phones) for health information-seeking among Hispanics.^15^ However, these data do not reflect the specific contexts of Hispanics who are residing in more recent settlement areas and in rural communities, where they may experience distinct circumstances and barriers to both technology and health resources as compared with Hispanics in more established and urbanized areas. A better understanding of technology utilization in relation to healthcare access is needed to determine the appropriateness and feasibility of technology-based approaches for improving health information access and healthcare services among this emerging population, particularly in new settlement areas such as South Carolina (SC).^19^

In 2010, staff at the *Clínica El Buen Samaritano*/Good Samaritan Clinic, a Hispanic-serving free medical clinic in the SC Midlands, identified an information gap regarding access to technology and health information-seeking practices among local Hispanics. Given the lack of relevant local data, clinic staff obtained funding from Blue Cross/Blue Shield of SC to assess local practice patterns. They then engaged the SC Hispanic/Latino Health Coalition to conduct this research.

## Instrumentation

The principal investigator (D.K.H.M.) developed a survey instrument with input and feedback from Good Samaritan clinic staff. The original instrument consisted of 54 items, which were organized into four sections: Access to Healthcare Services (4 items); Sources of Health and Healthcare Information (7 items); Technology and Health (18 items); and Demographics (25 items). The survey protocol was flexible in that not all participants were asked all questions. For example, if a participant answered “no” to questions regarding access/ownership of a cell phone or computer, the interviewer skipped the follow-up questions regarding cell phone or computer usage. Two native Spanish speakers translated the original English-language survey into Spanish; subsequently, two other bilingual speakers back-translated the Spanish language version into English.^20^ The principal investigator then compared the original and back-translated versions, which resulted in minor editorial changes to the survey. Before initiating data collection, the survey was pilot-tested with native Spanish speakers to assess the flow of the presentation and estimate administration time. To broaden the geographic representation of Hispanics, we conducted a second round of surveys between January 2015 and May 2016 beyond the Columbia Metropolitan area. As a result, we made minor changes to the survey, eliminating a question regarding knowledge of and access to the *Clínica El Buen Samaritano*, resulting in a 53-item survey.

## Methods

The University of South Carolina Institutional Review Board reviewed and approved the human subject protocol. Between September and December 2011, the principal investigator recruited and trained 10 bilingual research assistants through the SC Hispanic/Latino Health Coalition and a university student group affiliated with the local Hispanic-serving free medical clinic. All research assistants completed a formal in-service training, conducted by the principal investigator, which addressed the principles of informed consent and voluntary participation and the research study protocols. Data collectors were responsible for contacting and recruiting potential participants and for conducting individual, oral, interviewer-administered surveys. Participants did not receive incentives.

Research assistants conducted 216 survey interviews, lasting 10 and 30 min, between September and December 2011. Data collection occurred in the Columbia Metropolitan Area (i.e., Richland and Lexington Counties). We accessed participants through the Good Samaritan Clinic (*n*=131), local Hispanic-serving churches providing English-as-a-Second Language classes (*n*=29), and a *Consulado Movíl de México* (Mexican Mobile Consulate) event held on a Saturday at a public school in Lexington, SC (*n*=56). The *Consulado Móvil* is a traveling assistance program established by the Mexican *Cónsul General* in Raleigh, North Carolina^21^ (NC), that offers specific consular services (e.g., Mexican passports, consular identification cards) at rotating locations throughout NC and SC, reducing costs in travel and time for Mexican nationals who require consular assistance.

In 2015/2016, we administered 145 surveys, recruiting participants through Hispanic-serving churches and businesses (*n*=42) in a more diverse geographic area, including Beaufort, Chesterfield, Colleton, Lancaster, Richland, and Saluda Counties. The majority of participants (*n*=103) were accessed at three *Consulado Móvil* events held in Columbia, SC (*n*=46), Ridgeland, SC (*n*=27), and Monroe, NC (*n*=30), a site that served SC Hispanics living along the NC/SC border. Similar to data collection in 2011, the Research Assistants administered the survey orally to the majority of respondents. However, given changes in the *Consulado Móvil* processes in 2015/2016, potential respondents often did not have the time to participate in an orally administered survey. As a result, the research team provided some participants with the option of a self-administered paper survey, which they could complete as they moved through the lines and waiting rooms of the consular process. On completion, they returned the completed survey to the research assistant. We used the SAS 9.3 statistical analysis program (SAS Institute, Cary, NC) to manage the data and to compute descriptive data analysis. For reporting purposes, we translated responses to open-ended questions from Spanish to English; these are presented in italics.

## Findings

A total of 361 self-identified Hispanics, aged 18 or older, and residing in SC responded to the survey (Table 1). In terms of gender, age, and birth place, participant demographics were similar for both time periods.

**Table 1.** **Participant Demographics**

	2011 (*N*=2016), *N* (%)	2015–2016 (*N*=145), *N* (%)
Country of origin		
Mexico	155 (72.0)	119 (83.3)
Guatemala	22 (10.0)	4 (2.5)
Honduras	19 (9.0)	7 (4.5)
El Salvador	4 (2.0)	0
Cuba	3 (1.0)	0
Venezuela	2 (1.0)	0
Dominican Republic	1 (0.5)	1 (0.6)
Panama	1 (0.5)	0
US Mainland	3 (1.0)	14 (9.0)
Puerto Rico	6 (3.0)	0
Mean age		
	36.5	33.5
Gender		
Male	90 (42)	65 (45)
Female	126 (58)	80 (55)
Education		
Mean (range)	8.4 years (0 to >16)	8.9 years (0 to 18)

We assessed both language utilization (i.e., language spoken at home and work) and language preference for health information. The clear majority 82% (*n*=179) of 2011 participants reported speaking only Spanish at home, 15% (*n*=33) reported speaking both English and Spanish at home, and only 1% (*n*=3) spoke only English at home. In comparison, 70% (*n*=101) of the 2015/2016 participants indicated Spanish as the language of preference at home. In terms of language preference for receiving health information, 90% of the 2011 participants indicated Spanish, with 7% indicating a preference for English language health information and 3% preferring health information in both Spanish and English. In contrast, among the 2015/2016 participants, 72% preferred Spanish, with 30% indicating a preference for health information either in English or in both Spanish and English. Table 2 summarizes the participants' language preferences, health status, and health service utilization.

**Table 2.** **Self-Reported Language Preferences, Health Status, and Health Service Utilization**

	2011 (*N*=216), *N* (%)	2015/2016 (*N*=145), *N* (%)
Language preference at home		
Spanish only	179 (82)	101 (70)
English only	3 (1)	5 (3)
Spanish and English	33 (15)	37 (25)
Another language	0	2 (1)
Language preference for health information		
Spanish only	90	72
English only	7	15
Spanish and English	3	13
How would you describe your current health?		
Poor	32 (15)	10 (7)
Fair	64 (30)	51 (35)
Good	91 (42)	48 (33)
Very good	14 (6.5)	22 (15)
Excellent	14 (6.5)	14 (10)
Have you used SC medical services in the past year?		
Yes	93 (43)	83 (56)
What type of services did you use? (Note: Participants could report using more than one type of service)		
Community clinic	44 (20)	29 (19)
Private doctor's office	19 (8.5)	33 (21)
Hospital emergency room	41 (19)	25 (16)
Public health department	9 (4)	11 (7)
Urgent care clinic	20 (9)	11 (7)
Free clinic	74 (34)	7 (4.5)
Mental health center	4 (2)	1 (>1.0)
Pharmacy	0	31 (20)
Traditional healer	1 (>1.0)	1 (>1.0)

SC, South Carolina.

**FIG. 1.**
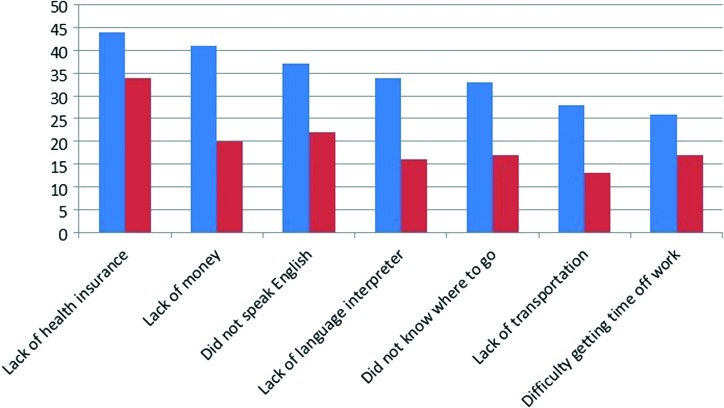
Self-reported healthcare services access barriers: 2011 (*blue*) and 2015/2016 (*red*).

We also assessed self-reported healthcare services access barriers, presented in Figure 1.

### Access to technology devices and internet service

Survey respondents reported a high level of personal access to a cell phone, at 89% in 2011 and 96.6% in 2015/2016, and there were no notable gender differences in cell phone utilization rates at either time (Table 3). However, reports of having a computer in the home decreased slightly from 2011 (*n*=125; 58%) to 2015/2016 (*n*=78, 53.9%), although a higher percentage of 2015/2016 participants reported knowing how to use a computer and having more access to computers outside the home, most commonly a public library. Internet usage trends also increased, with 74% of 2011 participants reporting use of the internet “several times a week” or daily, compared with 90% in 2015/2016. Findings from 2015/2016 indicated nearly double the percentage of respondents reporting daily internet access, markedly higher access to Facebook, and a much lower proportion of respondents using the internet only a couple of times a month.

**Table 3.** **Information Technology Access and Utilization**

	2011 (*N*=216), *N* (%)	2015/2016 (*N*=145), *N* (%)
Do you have access to a cellphone?		
Yes	193 (89)	140 (97)
Do you send and receive text messages by cell phone?		
Yes	143 (89)	134 (92)
How often do you send or receive text messages by cell phone?		
Only a couple times a month	32 (21)	16 (12)
Several times a week	62 (42)	35 (26)
Every day	55 (37)	84 (62)
Do you know how to use the computer?		
Yes	103 (48)	90 (62)
Are there other people in your household/family who know how to use the computer?		
Yes	151 (70)	112 (77)
Do you have a computer in your home?		
Yes	125 (58)	78 (54)
Do you have access to a computer at a location other than your home?		
Yes	66 (30)	66 (46)
How often do you use the internet?		
Only a couple times a month	33 (26)	14 (10)
Several times a week	50 (40)	37 (26)
Every day	43 (34)	93 (64)
Do you use email?		
Yes	96 (44)	89 (61)
If yes, how often do you use email?		
Only a couple times a month	28 (29)	22 (24)
Several times a week	44 (46)	30 (33)
Every day	24 (25)	37 (42)
Do you use Facebook?		
Yes	90 (42)	106 (73)
If yes, how often do you use Facebook?		
Only a couple times a month	12 (13)	14 (13)
Several times a week	49 (54)	30 (28)
Every day	29 (32)	62 (59)

### Patterns of internet usage for health information

**FIG. 2.**
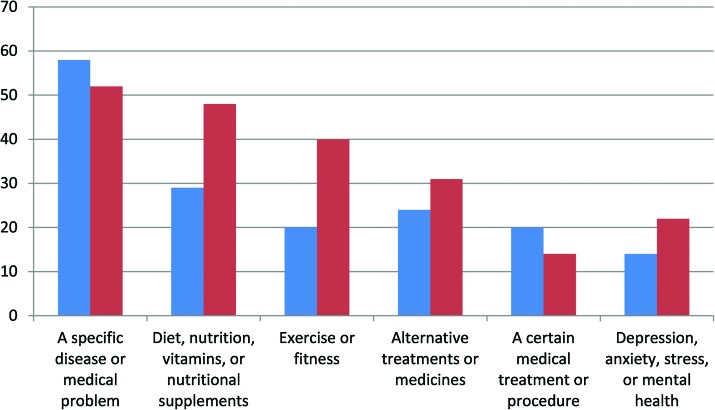
Most commonly reported topics of health information-seeking on the internet: 2011 (*blue*) and 2015/2016 (*red*).

In terms of receptivity to accessing health information via technology, the majority of respondents in both time periods indicated that they would “like to receive health information by cell phone.” Convenience, simplicity, and ease of access were the most frequently reported reasons associated with cell phones (i.e., “cellphones are simple and fast,” “I always have my cellphone with me.”). Respondents who acknowledged having searched the internet for health information were asked to assess the trustworthiness of the health information obtained on the internet; findings from both time periods were similar. The majority reported that they thought the internet was a “good source of health information.” Representative responses related to accessibility, ease, immediacy, and responsiveness included “The internet is fast and easy to find information”; “The internet has information about whatever you want to know”; “You can get immediate advice about your heath without going to the doctor”; and “You can ask questions about particular disease or illness.” It is important to note that a substantial number of participants in both time periods questioned the trustworthiness of internet health information (32% in 2011; 39.4% in 2015/2016). Among those reporting access barriers or lack of trust in health information available on the internet, responses reflected lack of utilization knowledge (e.g., “I don't know how to use the internet”), difficulty understanding the information, and distrust (e.g., “Information on the internet can be propaganda or advertising”). Table 4 presents findings related to health information-seeking; Figure 2 summarizes the most commonly reported topics of health-related internet searches.

**Table 4.** **Health Information-Seeking Patterns**

	2011 (*N*=216), *N* (%)	2015/2016 (*N*=145), *N* (%)
What is your most trusted source of health information?		
Family member	42 (21)	47 (30)
Friend/neighbor	37 (18)	29 (19)
Coworker	2 (1)	12 (8)
Pastor/Person at church	9 (4)	18 (11)
Flyer or poster at a public place	3 (2)	9 (6)
Radio	5 (2.5)	9 (6)
Television	10 (5)	15 (10)
Newspaper	5 (2.5)	7 (4.5)
Internet	12 (7)	32 (20)
Doctor or other healthcare personnel	69 (34)	49 (31)
How often do you need help to read and understand written health information?		
Never	62 (29)	46 (32)
Sometimes	102 (47)	73 (50)
Always	52 (24)	26 (18)
How often do you find it difficult to understand the health information you receive from a doctor or other healthcare provider?		
Never	106 (49)	50 (34)
Sometimes	86 (40)	71 (49)
Always	24 (11)	24 (16)
Have you ever looked for health information on the internet?		
Yes	98 (45)	84 (58)
Do you think information on the internet is trustworthy?		
Yes	63 (68)	88 (61)
No	5 (5)	5 (5)
Not sure	25 (27)	36 (35)
Do you think the internet is a good source of health information for you or your family?		
Yes	180 (86)	102 (81)
Would you like to receive health information through your cellphone?		
Yes	156 (72)	97 (70.3)
In which language have you looked for health information on the internet?		
Spanish	75 (76)	57 (60)
English	9 (9)	17 (18)
Both	15 (15)	21 (22)

## Discussion

These findings reflect national trends in Hispanic technology adoption, including cellphone ownership, desktop/laptop computer ownership, and internet use.^15^ A limitation to our findings is that the survey did not discriminate between cellphones and smartphones, as smartphones were not as available in 2011. However, the high rate of cellphone ownership among this sample of relatively young, not-highly-educated Hispanics, the vast majority of whom were immigrants, is similar to national trends of increasing Hispanic cellphone ownership (from just more than 80% in 2011 to 92% in 2015/2016) and declines in desktop/laptop ownership (from 88% in 2011 to 78% in 2015/2016).^22^ Survey responses indicated an increasing proportion of cell phone sending or receiving text messages via their cell phones. These findings suggest that Spanish-language text messaging may be an effective way to disseminate certain types of health information among this population. For example, text messaging may be an effective and appropriate medium for appointment or immunization reminders or notices regarding health fairs or specific services.

In terms of access to computers and internet technology, although reported computer ownership was lower in 2015/2016, internet access was 90%, a marked increase from 2011. This finding reflects national trends of increasing internet accessibility, especially via cellphones and smartphones. Although participants in 2015/2016 were somewhat more likely to conduct health information internet searches in English, the vast majority reported seeking information in Spanish. Clearly, the demographic factors of this population must be taken into consideration regarding the accessibility of health information. Given the reported educational level and Spanish language preference among this sample of Hispanics, healthcare providers and health educators must address the availability of Spanish language information geared to a population with relatively low levels of limited literacy level of Spanish language information. Systematic, community-wide collaborative efforts to provide Hispanics with limited English proficiency both internet access and Spanish language health information are clearly warranted.

Furthermore, as these findings imply, healthcare providers need to better understand the complexities underlying Hispanics' access to and trust of health information sources. The majority of participants indicated *doctor* as the *most trusted* source of health information. However, given that more than 40% of the participants reported not having any contact with the formal healthcare system in the past year, the opportunity or ability to access professionals (e.g., physicians, nurses, pharmacists) for health information among this more recent SC Hispanic immigrant population is likely to be limited. Particularly for those marginalized by language, immigration status, geography, and socioeconomic status, increasing the availability of culturally and linguistically appropriate and tailored health information through a variety of sources is needed to address existing health disparities and to promote health equity.

## Health Equity Implications

The achievement of health equity, defined as the “attainment of the highest level of health for all people,”^23^ requires attention to biological, physical, social, cultural, and environmental factors that impact the health status of underserved communities and racial and ethnic minorities.^24^ Equal recognition and valuing of the health needs and potential of all groups requires focused and ongoing societal efforts to address avoidable inequalities, historical and contemporary injustices, and the elimination of health and healthcare disparities. The development and implementation of culturally and linguistically tailored interventions and programs exemplify efforts to address these disparities and promote health equity.^25^ Nationally, patients who experience access barriers are more likely to report relying on the internet to obtain health information, highlighting the potential health equity impact by reliable, accurate, and culturally and linguistically tailored healthcare information for vulnerable population groups, including Hispanics in more recent settlement areas.^26–28^ Health information access increases patient knowledge and ability to access healthcare services. Therefore, it is imperative to provide healthcare information through trusted and readily available sources, particularly among Hispanics without regular access or contact with the US healthcare system. Schools, churches, and adult education classes in English as a Second Language could include this type of information in their curricular activities. Healthcare providers should also monitor the quality of Spanish-language text messages, emails, and list serves or blogs.

Providing culturally and linguistically appropriate health information through accessible technology is one approach for healthcare providers and organizations to address health literacy and enhance appropriate and timely healthcare access among minority and populations with limited English proficiency. Further research is warranted to identify best practices for utilizing communication technologies to enhance access to healthcare information and services among hard-to-reach populations. Ongoing efforts by policymakers, researchers, clinicians, educators, librarians, and community outreach workers to enhance access to healthcare information and services are essential for achieving health equity. Clinicians and researchers who utilize and study technology-based approaches to improving access to healthcare information and services should be cognizant that efforts to further health equity are complex and need to address a wide range of social, cultural, educational, and economic factors.

## Abbreviations Used

NC: North Carolina
SC: South Carolina
